# Supplementary material for: Summer atmospheric circulation over Greenland in response to Arctic amplification and diminished spring snow cover
Source: Nat Commun. 2023 Jun 23;14:3759. doi: 10.1038/s41467-023-39466-6 (PMC10290063; doi:10.1038/s41467-023-39466-6)
Supplement: Supplementary file 1 — Supplementary Information [file 41467_2023_39466_MOESM1_ESM.pdf]

Supplementary Table 1 Long-term (1979-2022) seasonal trends in the mean and select percentiles of S_{ag}. Percentiles computed from daily S_{ag} for each season and year.

	Statistic	Slope (SD decade^{-1})	P-value
**MAM**	Mean	-0.011	0.93
	25th %tile	-0.005	0.90
	50th %tile	0.015	0.69
	75th %tile	-0.0018	0.96
	90th %tile	-0.0060	0.90
**JJA**	Mean	0.29	0.014
	25th %tile	0.096	0.009
	50th %tile	0.10	0.013
	75th %tile	0.11	0.017
	90th %tile	0.10	0.081
**SON**	Mean	0.086	0.48
	25th %tile	0.043	0.15
	50th %tile	0.040	0.28
	75th %tile	-0.012	0.76
	90th %tile	-0.032	0.52
**DJF**	Mean	0.051	0.67
	25th %tile	0.017	0.72
	50th %tile	0.036	0.52
	75th %tile	0.026	0.68
	90th %tile	0.016	0.81

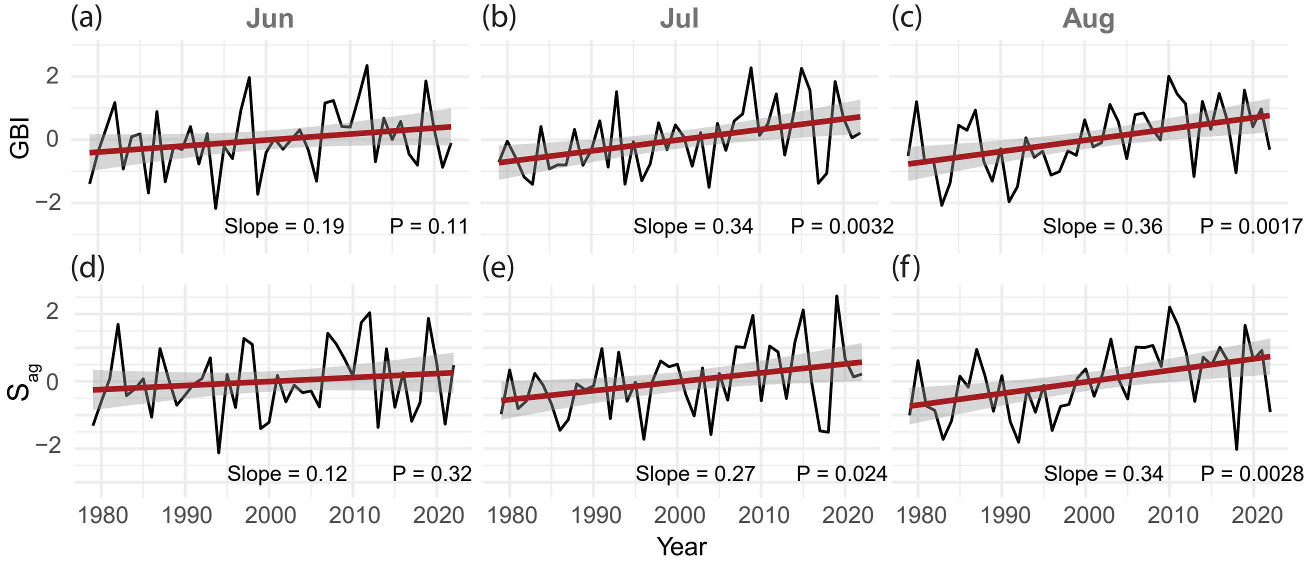

Supplementary Fig. 1 Linear trends in Summer North Atlantic atmospheric circulation broken down by month. Top row shows the 1979-2022 linear trend in **a**, June, **b**, July, and **c** August monthly-mean GBI. Bottom shows the 1979-2022 linear trend in **d**, June, **e**, July, and **f** August monthly-mean S_{ag}. Each panel is annotated with the slope of the line [SD per decade] and the corresponding p-value.

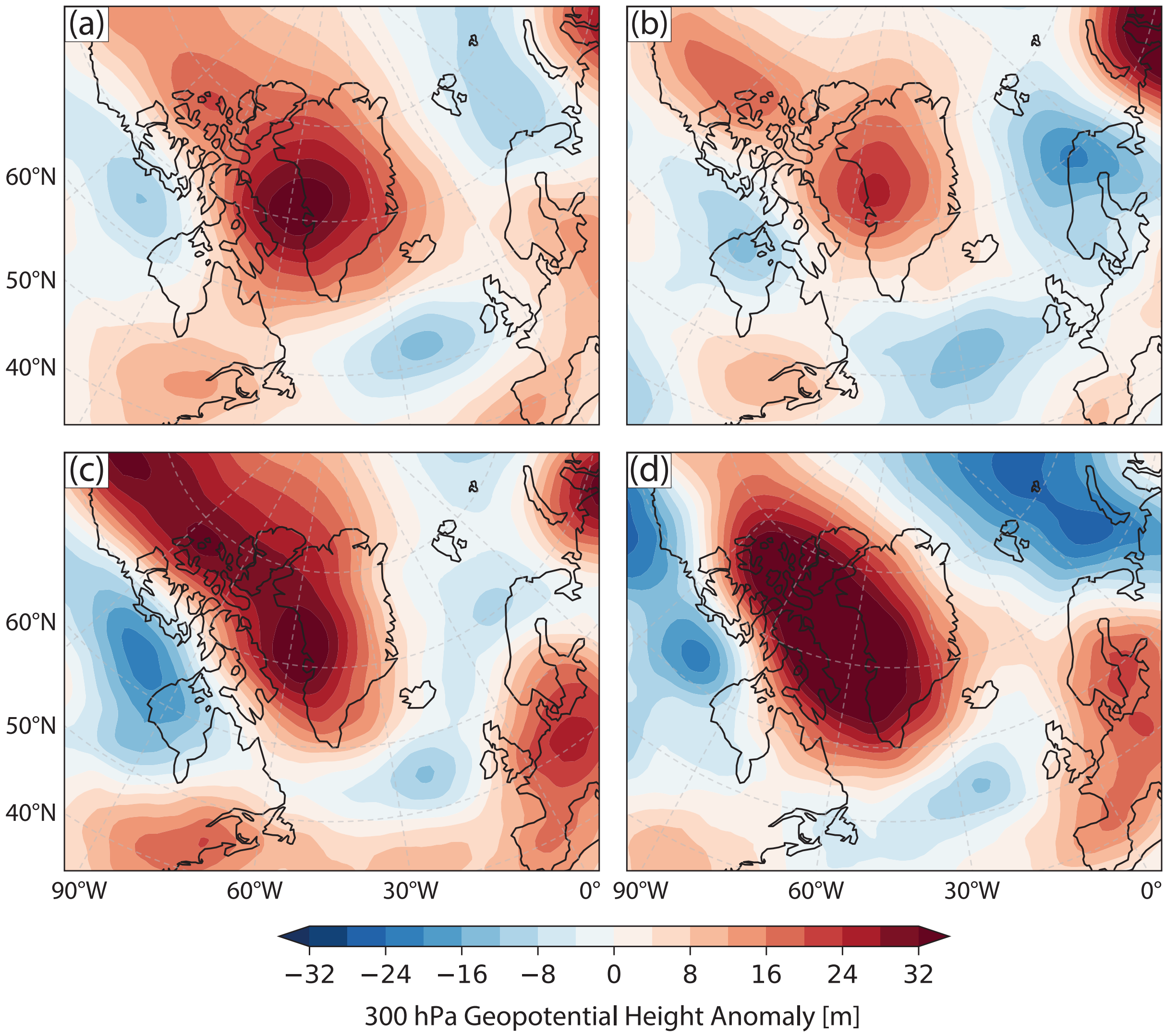

Supplementary Fig. 2 Interdecadal change in the July atmospheric response to low May North American SCE area. Panels show the regression coefficients relating July Z300 to antecedent May North American SCE area over **a**, the full 1979-2022 study period, **b**, 1981-2000, **c**, 1991-2010, and **d**, 2011-2020.

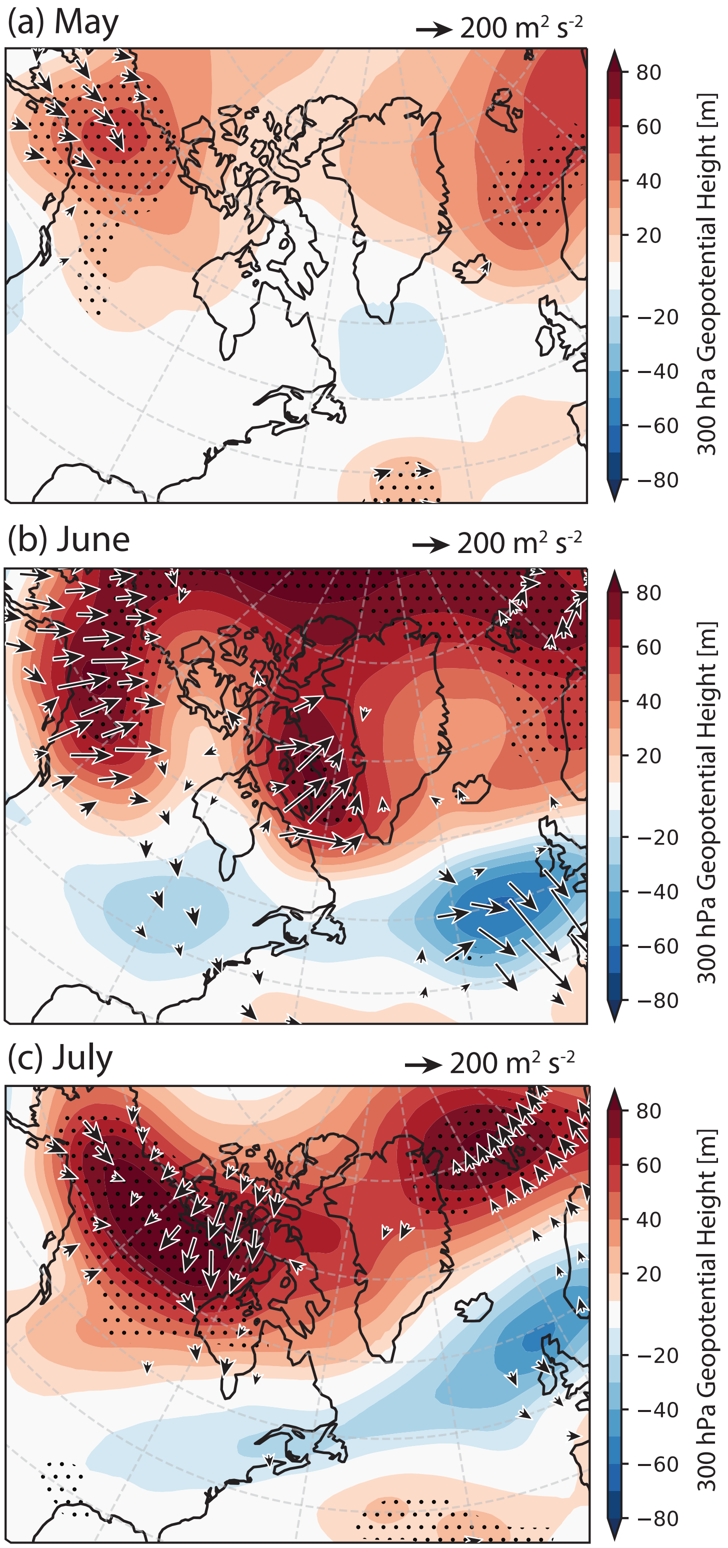

Supplementary Fig. 3 Atmospheric response to low May North American snow cover as modeled by the Community Earth System Model 2. Shading shows composite mean differences in **a,** May, **b**, June, and **c**, July 300 hPa geopotential height between a 10-year control simulation and a prescribed snow simulation in which snow cover was reduced to zero over the whole of North America on May 1st of each year. Vectors show the anomalous horizontal wave activity flux as calculated from the composite mean 300 hPa height differences shown by the shading. Stippling indicates significant differences at the 95% confidence level according to a paired t-test. Hypothesis tests performed individually for each grid cell without multiple testing or spatial autocorrelation correction.

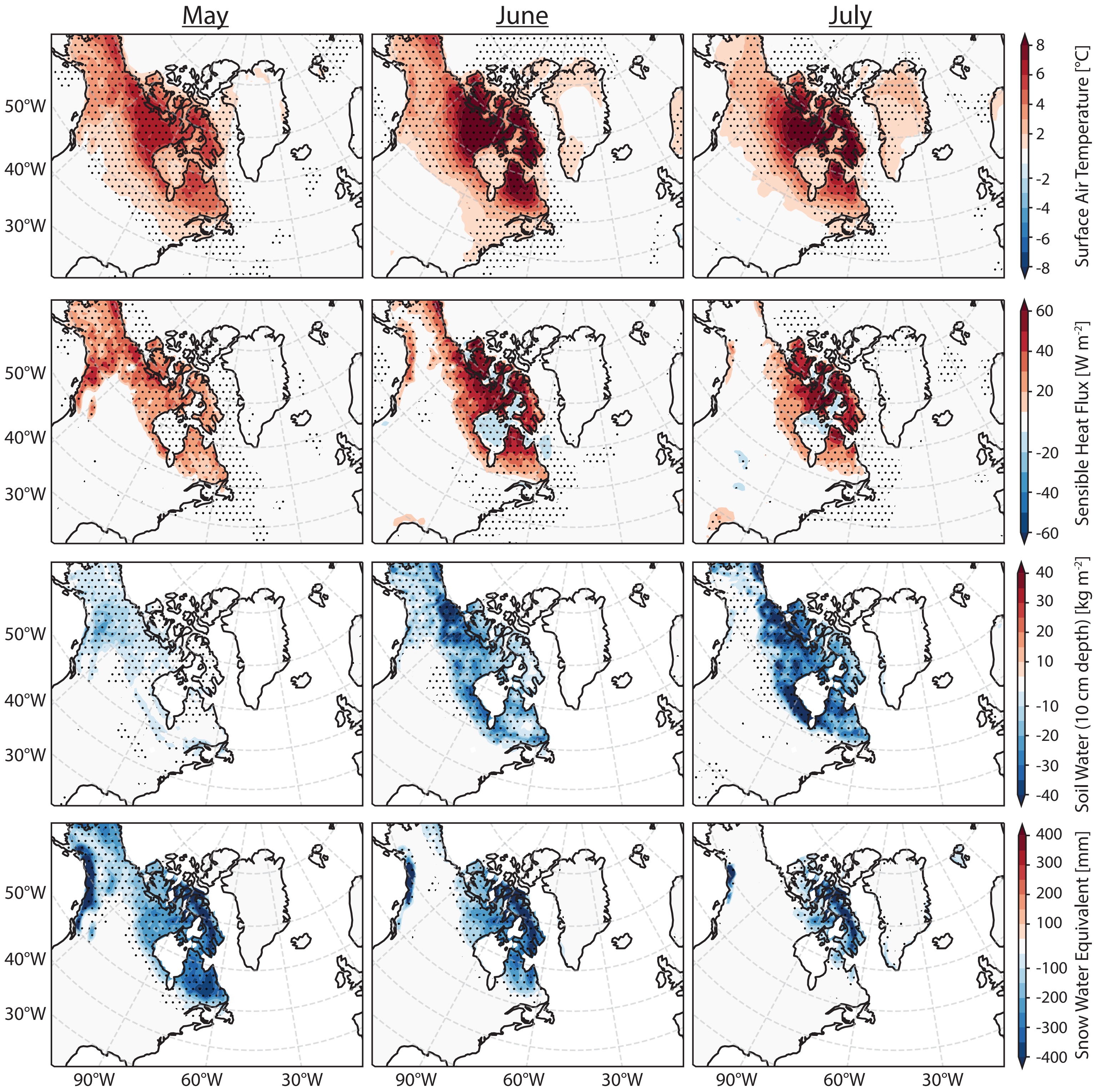

Supplementary Fig. 4 Surface response to low May North American snow cover as modeled by the Community Earth System Model 2. Shading shows composite mean differences in (columns) May, June, and July surface variables between a 10-year control simulation and a prescribed snow simulation in which snow cover was reduced to zero over the whole of North America on May 1st of each year. Variables are organized by row as labeled to the right. Stippling indicates significant differences at the 95% confidence level according to a paired t-test. Hypothesis tests performed individually for each grid cell without multiple testing or spatial autocorrelation correction.

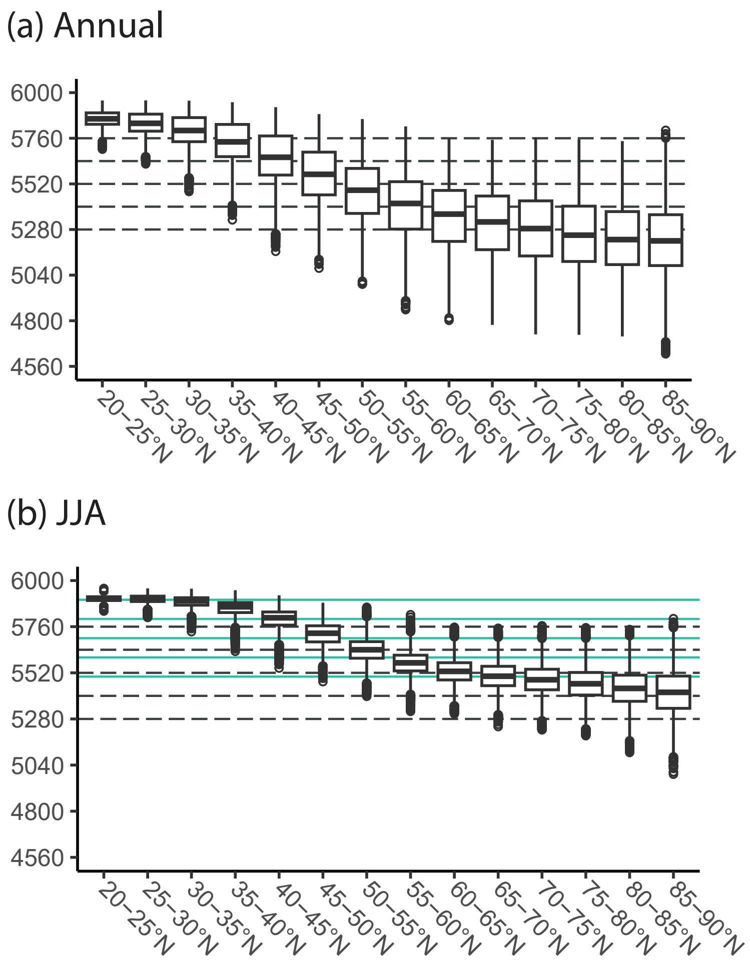

Supplementary Fig. 5 Climatology of Northern Hemisphere Z500 for (a) all days (b) JJA days during the 1979–2022 study period. Boxplots summarize daily zonal means grouped into 5° latitudinal bins spanning the midlatitudes as labeled along the x-axis. Dashed horizontal lines mark aggregate sinuosity isohypse values used in ref[22]. Solid green horizontal lines in (b) mark JJA S_{ag} isohypses used here.

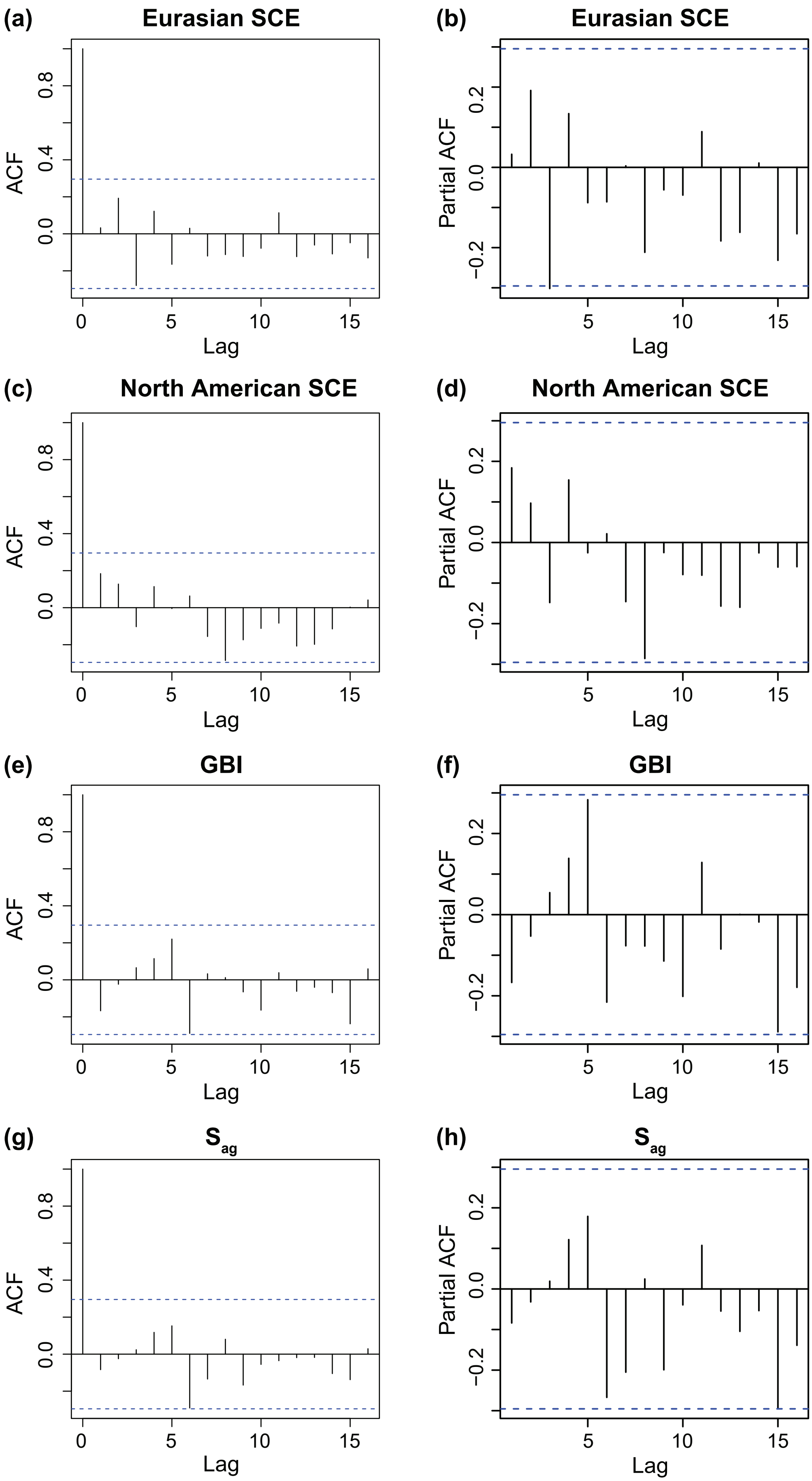

Supplementary Fig. 6 Autocorrelation (**a, c, e, g**) and partial autocorrelation function (**b, d, f, h**) plots for (**a-b**) June Eurasian SCE area, (**c-d**) June North American SCE area, (**e-f**) JJA GBI, and (**g-h**) JJA S_{ag}.

Supplementary Table 2 Durbin-Watson test results. The tested null hypothesis is that the true autocorrelation of each of the annual time series (i.e., without taking the 5-year running mean) presented in Fig. 2a is equal to zero.

**Time Series**	**DW Statistic**	**P-value**
June Eurasian SCE	1.93	0.35
June North American SCE	1.62	0.07
JJA GBI	2.31	0.81
JJA S_{ag}	2.14	0.62

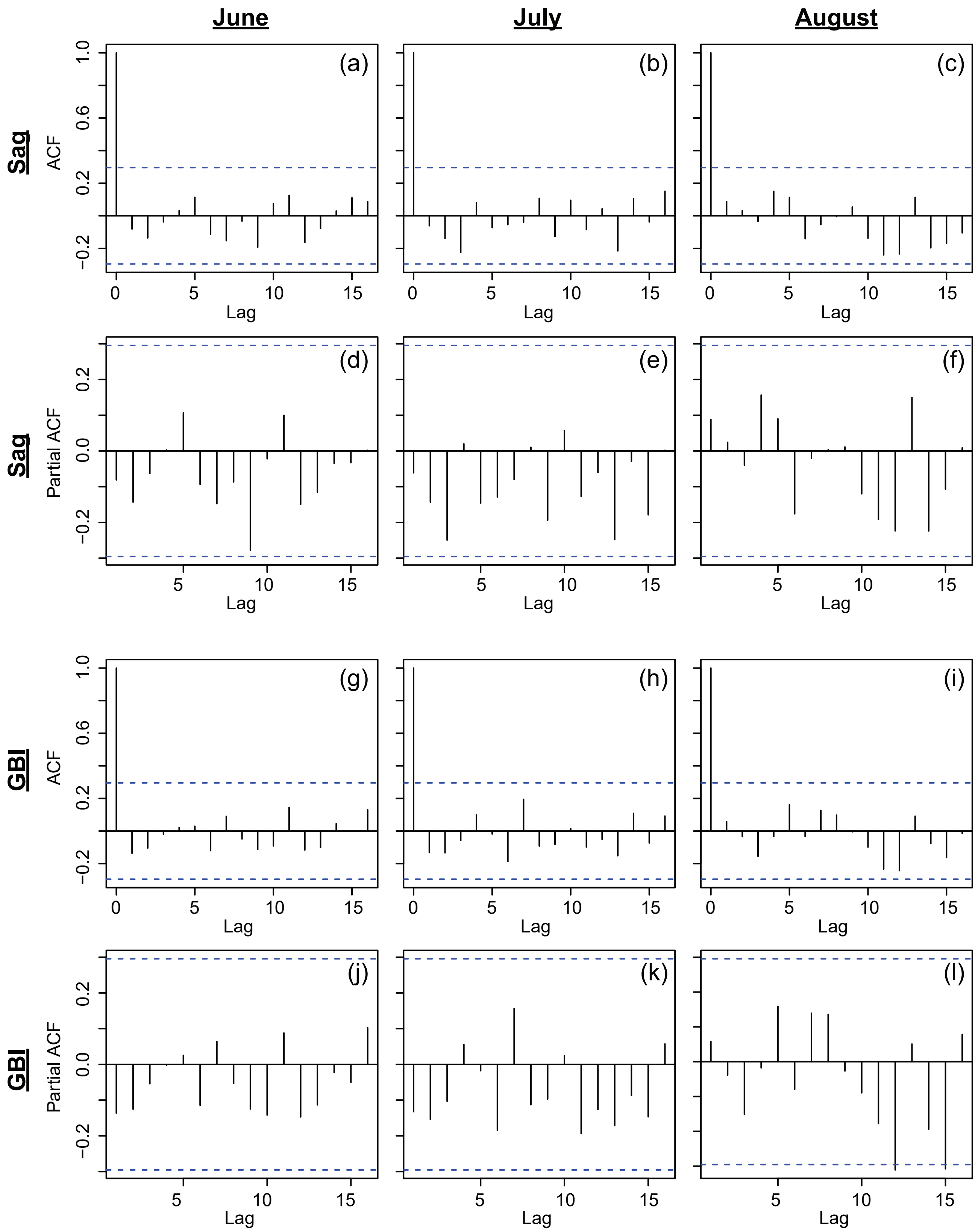

Supplementary Fig. 7 Autocorrelation (**a-c**, **g-i**) and partial autocorrelation function (**d-f**, **j-l**) plots for monthly-mean time series of (**a-f**) Sag and (**g-l**) GBI for the months of June (left column), July (middle column), and August (right column).
